# A novel monoclonal antibody against the N-terminus of Aβ_1-42_ reduces plaques and improves cognition in a mouse model of Alzheimer’s disease

**DOI:** 10.1371/journal.pone.0180076

**Published:** 2017-06-29

**Authors:** Hai-Yan Xing, Bin Li, Dan Peng, Chun-Yan Wang, Guan-Ying Wang, Pan Li, Ying-Ying Le, Ji-Ming Wang, George Ye, Jian-Hong Chen

**Affiliations:** 1 Department of Pharmacy, Daping Hospital & Research Institute of Surgery, Third Military Medical University, Chongqing, China; 2 Anogen-Yes Biotech, Mississauga, Ontario, Canada; 3 Institute for Nutritional Sciences, Shanghai Institutes for Biological Sciences, Chinese Academy of Sciences, University of Chinese Academy of Sciences, Shanghai, China; 4 Cancer and Inflammation Program, Center for Cancer Research, National Cancer Institute at Frederick, Frederick, Maryland, United States of America; Nathan S Kline Institute, UNITED STATES

## Abstract

Senile plaques consisting of Amyloid-beta (Aβ) peptides, in particular Aβ_1–42_, are the hallmark of Alzheimer’s disease (AD) and have been the primary therapeutic targets. Passive immunotherapy with monoclonal antibodies (mAbs) has shown initial success in mouse models of AD. However, the existing Aβ-directed mAbs mostly were tested on animal models or patients with advanced disease. The effects and mechanisms of mAbs on animals or human trial participants in the prodromal phase of AD are not fully clarified. In the current study, a novel mAb (3F5) directed against the 1–11 amino acids of Aβ_1–42_ was generated by immunizing mice with an emulsion of full length human Aβ_1–42_. The mAb (3F5) showed the ability to disrupt Aβ_1–42_ aggregation and prevent Aβ-mediated neurotoxicity *in vitro*. In a mouse model of AD, administration with 3F5 for 3 months in 6 months-old mice demonstrated that the mAb specifically bound with Aβ_1–42_ to promote the depolymerization of Aβ fibrils, facilitated endocytosis of Aβ_1–42_ by microglia, and attenuated the death and apoptosis of neuronal cells, accompanied by neurite outgrowth. APP/PS1 double-transgenic mice treated with 3F5 mAb showed reduced memory loss, cognitive decline, and decreased levels of amyloid deposits in the brain. Aβ_1–42_ levels in cerebral tissues were also significantly reduced, whereas serum Aβ_1–42_ was markedly increased. Interestingly, the concentration of 3F5 in peripheral circulation is much higher than that in the brain. These results indicate that 3F5 is able to cross the blood-brain barrier (BBB) to bind Aβ and initiates the phagocytosis of antibody/Aβ complexes by microglia in the amyloid depositing mice. 3F5 also promotes Aβ efflux from the brain. As a consequence, the antibody reduces plaques in the AD mouse brain, in association with reduction in the pathology of AD.

## Introduction

Alzheimer's disease (AD), a progressive neurodegenerative disorder, is characterized by memory and cognition impairment. Strong genetic and biochemical evidence highlights β-amyloid deposition in the brain as a causative factor for this neurodegeneration cascade [1]. Thus, approaches to reducing Amyloid-beta (Aβ) production or remove Aβ_1–42_ deposits are actively explored as therapeutic modalities for AD [2]. Among these approaches, immunotherapy is considered as the first choice of disease-ameliorating treatment.

Passive immunization, treating patients with ex vivo produced monoclonal antibodies (mAbs) against Aβ peptides, has become potentially an effective strategy to prevent or treat AD [3]. Preclinical studies have shown that peripheral or intracerebroventricular injection of anti-Aβ antibodies reduces Aβ levels in the brain and improves cognitive function [4, 5]. Furthermore, antibodies against the N-terminus of Aβ_1–42_ may be superior to those against the middle and C-terminus Aβ_1–42_ segments to eliminate Aβ plaques [6]. However, global phase III trials of bapineuzumab, a mAb specific for the N-terminus of Aβ_1–42_ peptide, showed lack of efficacy on clinical endpoints in patients with mild to moderate AD [7]. A commonly held view is that the regimen of immunotherapy applied may be too late to halt or reverse the disease course. Consequently, there is a need to re-evaluate current immunotherapy strategies in terms of timing, safety and efficacy.

In the current study, a novel mAb (3F5) directing at the N-terminal 1–11 peptide region of Aβ_1–42_ was generated by immunizing mice with an emulsion of full length human Aβ_1–42_. This mAb demonstrates the capacity to disrupt Aβ_1–42_ aggregation *in vitro* and reduce Aβ-mediated neurotoxicity. *In vivo*, in 6 month old APP/PS1 double-transgenic mice (Tg-mice), 3F5 crosses the blood-brain barrier (BBB) to bind Aβ to promote the phagocytosis of antibody/Aβ complex by microglia. 3F5 also initiates Aβ efflux from the brain. Thus, when given mice at an early stage of AD, 3F5 is able to markedly alleviate the progress of AD.

## Materials and methods

### Chemicals and reagants

Synthetic Aβ fragment peptides 1–11, 12–28, 25–35 and 33–42 were purchased from Bachem Biotechnology (Bachem, Switherland). Aβ_1–42_ was purchased from American Peptide Company (California, USA). Non-specific IgG (#A7028), HRP-conjugated secondary antibody (#A0216) and Propidium Iodide (PI) (#ST512) were obtained from Beyotime Biotechnology (Shanghai, China). Goat serum (#SL038), β-mercaptoethanol (#M8210) and Fluorescein Isothiocyanate Isomer I (FITC) (#F8070) were purchased from Solarbio Life Sciences (Beijing, China). Thioflavin T (ThT) (#T3516), Methyl Thiazolyl Triumvirate (MTT) (#M2128), Dimethyl Sulfoxide (DMSO) (#472301), All-trans-retinoic Acid (RA) (#R2625), Paraformaldehyde (#16005) and Diaminobenzidine (DAB) (#DA1010) were purchased from Sigma—Aldrich (USA). All other reagents were from Sigma-Aldrich, unless otherwise indicated.

### The development of mAb clone 3F5

The hybridoma of anti-Aβ_1–42_ mAb clone 3F5 was generated through intraperitoneal immunization of 6–7 week old Balb/C mice with an emulsion of full length human Aβ_1–42_ (Sequece: DAEFRHDSGYEVHHQKLVFFAEDVGSNKGAIIGLMVGGVVIA) and Complete Freund’s Adjuvant (Sigma, F5881). The immunization was boosted three times intraperitoneally with full length human Aβ_1–42_ in incomplete Freund’ Adjuvant (Sigma, F5506). The spleen of the immunized mouse was isolated and the cells were dispersed with a 200mu mesh under sterile condition. The spleen cells were mixed with the myeloma cell line Sp2/0-Ag14 (ATCC, CRL-1581) at a 10:1 ratio, while fusion reagent Polyethylene Glycol 1500 solution (Roch 783641) was added dropwise into the mixture. After plating the cell mixture to microwell plates, the hybridomas were selected by adding HAT Supplement (Gibco, 31062–011) to the medium. Anti-Aβ_1–42_ antibody secreting clones were screened and subcloned based on the reaction in full length human Aβ_1–42_ coated ELISA plates. The isotype of the clone 3F5, as determined using the Mouse Typer Isotyping Kit (Bio-Rad, 17–2055), is IgG2b Kappa. The antibody was purified using Gammabind Plus Sepharose (GE Healthcare, 17-0886-02). ELISA plates coated with different human Aβ peptides were used to analyze the reactivity of mAb clone 3F5.

### Measurement of antigen-antibody affinity and specificity

Synthetic Aβ_1–42_ (100 μL, American Peptide Company, California, USA) (100 ng/mL) was added to the wells in 96-well ELISA plates (Corning, USA). The samples were coated at 4°C overnight. The plates were washed and blocked using 5% bovine serum album (BSA) in 5% CO_2_ atmosphere at 37°C for 2 h. Samples were then naturally dried for future use. Thereafter, synthetic Aβ_1–42_ (1 μg/mL) without conjugation was diluted (1, 0.5, 0.25, 0.125, 0.0625, 0.03125 and 0 μg/mL) and then pre-incubated with 3F5 (0.05 μg/mL) at 4°C overnight. The mixtures were added in Aβ_1–42_ pre-coated ELISA plates for 90 min at 37°C to measure the competitive binding by 3F5 with free Aβ_1–42_ or coated Aβ_1–42_. The optical density (OD) was detected at 495 nm on a microplate reader (BIO-RAD Model 2550 EIA Reader, USA).

Classical peptide mapping using binding ELISA was performed to identify the epitope of Aβ_1–42_ recognized by 3F5. The 96-well ELISA plates were coated with different Aβ_1–42_ fragments (aa1-42, aa1-11, aa12-28, aa25-35, aa33-42), and 3F5 was then incubated with Aβ_1–42_ fragments for 72 h at 37°C. 3, 3′, 5, 5′ -Tetramethylbenzidine (TEM) was added as a substrate in each well and optical density (OD) was detected at 450 nm on a microplate reader (BIO-RAD).

### Neurite outgrowth assay

Neurite outgrowth was examined according to previously described method [8]. Briefly, 2×10^3^ SH-SY5Y cells/well were seeded in a 24-well plate and cultured for 24 h at 37°C in a 5% CO_2_. 10 μM all-trans-retinoic acid (RA) were co-incubated with SH-SY5Y cells for 5 days followed by incubation with 10 μM Aβ_1–42_ fibrils. 3F5 antibody (10 μg/mL and 20 μg/mL) and IgG (10 μg/mL) were then added into the plate for an additional 24 h. The supernatant was discarded and 200 μL 4% paraformaldehyde was added to each well to fix the cells. Cell images were acquired by an inverted microscope (Olympus, Japan) and 10 fields (100×) were analyzed per group.

### MTT assay

SH-SY5Y neuroblastoma cells were obtained from the Cell Bank of Type Culture Collection of Chinese Academy of Sciences (Shanghai, China). Methyl thiazolyl triumvirate (MTT) assay was used to measure the ability of 3F5 to reduce the cytotoxicity of Aβ_1–42_ fibrils. Briefly, SH-SY5Y cells were grown on 96-well plates at a density of 4×10^5^ for 24 h. After treatment with the different concentrations of 3F5 (40 μg/mL, 20 μg/mL, 10 μg/mL and 5 μg/mL) and 10 μM Aβ_1–42_ for 48 h, the cells were incubated with 10 μL MTT (Sigma, 5 mg/mL in PBS). After incubation at 37°C for 4 h, supernatants were removed and 150 μL dimethylsulfoxide (DMSO) were added. The absorbance was monitored at 490 nm on a microplate reader (BIO-RAD).

### Propidium iodide assay

SH-SY5Y cells were seeded at 2×10^4^ per well in a 24-well plate in three replicates. Aβ_1–42_ fibrils at 10 μM were added into the wells to co-incubate with 3F5, IgG, or PBS for 48 h. The samples then were mixed with propidium iodide (PI, 50 μg/mL) and incubated for 15 min at room temperature in the dark. Cell images were visualized with blue filter in an inverted fluorescence microscope (IX70, Olympus, Japan).

### Annexin V-FITC / PI assay

The number of apoptotic and necrotic cells was counted using an Annexin V-FITC/PI staining kit (KeyGEN BioTECH, Nanjing, China) according to the manufacturer’s instructions. Briefly, SH-SY5Y cells were seeded in a 12-well plate at a density of 1×10^5^/well and then incubated in 5% CO_2_ atmosphere at 37°C for 48 h. Aβ_1–42_ at 10 μM was added in each well. Different concentrations of 3F5, IgG and PBS were added to the wells. IgG was considered as a negative control. SH-SY5Y cells were washed at 48 h later with cold PBS and collected by centrifugation at 92 g for 5 min. Subsequently, the cells were stained with a combination of fluorescein annexin V-FITC and PI for 10 min at room temperature. Apoptosis was examined with by flow cytometry (ACEA Biosciences, Santiago, USA).

### Morris water maze

Tg-mice were obtained from the Jackson Lab of the University of Western Ontario in Canada, and the wild-type (WT) mice were derived from the Animal Experimental Center of Zheng Zhou University. They were housed in a temperature- and humidity-controlled environment under a 12-h light/12-h dark cycle (lights on at 8:00) and had ad libitum access to food and water. The health and welfare of the animals were monitored at least once a day. Experiments were approved by the Animal Care and Welfare Committee of the Third Military Medical University. All animal were performed in accordance with the standard guidelines for the care and use of laboratory animals and were approved by the Biological Research Ethics Committee of Third Military Medical University. It has been stated that during the experiment period, if an animal experienced symptoms of torture (self-injury behavior, abnormal posture, and crying etc.) and rapid weight loss (more than 20% in several days), it would be euthanized, with sodium pentobarbital (120mg/kg). No mice were observed having such a condition in this study.

Tg-mice (females and males, 6 months) and WT littermates (males, 6 months) were divided into four groups (n = 6): Tg control, Tg 3F5, Tg IgG and WT control. 3F5-treatment or IgG-treatment mouse group was injected intraperitoneally with 0.5 g/kg of 3F5 or IgG twice a week until 9 months of age. The Tg-control and WT mouse groups were injected intraperitoneally with normal saline (NS) at equal volume with the treatment groups.

Morris water maze (MWM) test was used to investigate spatial learning and memory of mice. The diameter of swimming pool for MWM is 1.2 meters, and the temperature was maintained at 18–22°C. Time of detection was limited to 60 seconds, and the record was terminated when mice stayed on the platform for over 3 seconds. Mice were examined in three quadrants except for aim quadrant each day. After 5 days, the platform was removed for probe trial. Indicators included time of water maze latency, speed of swimming, frequency of platform-crossing and the time spent in target quadrant in behavioral testing.

### Thioflavin T assay

To measure the ability of 3F5 to disaggregate Aβ fibrils, synthetic Aβ_1–42_ was diluted to 10 μM with PBS and then incubated in a 96-well brown plate at 37°C for 48 h to form fibrils. Thereafter, 100 μL Aβ_1–42_ (10 μM) was co-incubated with different concentrations of 3F5 at 37°C for 24 h, 48 h or 72 h, followed by 10 μM thioflavin T (ThT) solution (50 mM phosphate buffer, pH 6.0) for 15 min in the dark at room temperature. Fluorescence was measured by Thermo Varioskan Flash (excitation at 450 nm and emission at 482 nm).

### Cytotoxicity

Synthetic Aβ_1–42_ dissolved in DMSO was diluted by carbonate buffer (CBS, pH 9.6) to 2.5 mg/mL, and FITC was added to shake for 2 h in the dark. Murine microglial cell line N9 (a kind gift from Dr. P. Ricciardi-Castagnoli, Universita Degli Studi di Milano-Bicocca, Milan, Italy) was grown in Iscove's modified Dulbecco's medium supplemented with 5% heat-inactivated FCS, 2 mM glutamine, 100 units/mL penicillin, 100 μg/mL streptomycin, and 50 μM 2-mercaptoethanol. The cells were plated at a density of 1×10^5^/well in a 24-well plate and incubated overnight. Aβ_1–42_ labelled with FITC (1 μg/mL) was mixed and co-incubated with 3F5 for 1 h at 37°C. The samples were added to the plates for co-incubation with N9 cells for 30 min in 5% CO_2_ at 37°C. Fluorescence was monitored by NovoCyte Flow Cytometer (ACEA Biosciences, Santiago, USA).

### Detection of Aβ_1–42_ and 3F5 in mouse cerebral tissues and peripheral blood

The concentration of Aβ_1–42_ and 3F5 in mouse cerebral tissues and peripheral blood was measured by ELISA (Elabscience BioTECH, Wuhan, China), according to the manufacturer’s instructions. All mice were quickly decapitated. The procedure was performed without anesthesia to avoid the potential effect of anesthetic drugs. Whole blood was collected from mice. Then, the cerebral tissues were cut into fragments and suspended in PBS for sonication. Thereafter, the samples were centrifuged for 10 min at 9200 g at 4°C. The supernatants were collected for detecting Aβ_1–42_ and 3F5. The optical density (OD) was obtained on the plate reader at 450 nm (BIO-RAD).

### Pathological staining and analysis for Aβ plaques and microhemorrhage

Immunohistochemistry was used to analyze the ability of 3F5 antibody to attenuate Aβ deposition in the brain of Tg-mice. 3 μm brain sections were mounted onto microscope slides and dried for 2.5 h. The sections were then soaked in sodium citrate buffer solution for 20 min in a microwave oven at low temperature for antigen retrieval. Sections were blocked with goat serum for 30 min and then were incubated with 3F5 (1: 20000) overnight at 4°C. The sections were washed with PBS and incubated with HRP-conjugated secondary antibody (1: 500) for 30 min at room temperature. Staining was visualized with diaminobenzidine and images were monitored by Upright Metallurgical Microscope (BX53M, Olympus, Japan).

Prussian blue histological staining was performed to assess microhemorrhage in cerebral tissues by previously described method [9]. Briefly, 3 μm brain sections attached to microscope slides were dewaxed and hydrated, then stained for hemosiderin in a solution containing 2% potassium ferrocyanide and 2% hydrochloric acid for 15 min. Thereafter, the sections were counterstained in a 1% Neutral Red solution for 10 min at room temperature. The sections were washed in distilled water, dehydrated and coverslipped with neutral balsam. The images of hemosiderin deposits were acquired by Upright Metallurgical Microscope (BX41, Olympus, Japan).

### Statistical analysis

All experiments were performed at least three times, and the results were expressed as the means ± standard deviation (SD). All data for each variable were checked for normality and homogeneity of variance before performing statistical analyses. The results were analyzed by one-way analysis of variance (ANOVA) followed by a SNK-q test for multiple comparisons. Repeated measures ANOVA followed by the least significant difference (LSD) test was used to compare the escape latencies among mouse groups. P<0.05 was considered statistically significant. Quantitative assessment of Aβ plaques area and the length of neurite were measured by ImageJ 1.48u.

## Results

### 3F5 specially binds to the N-terminus of Aβ_1–42_

**Fig 1:**
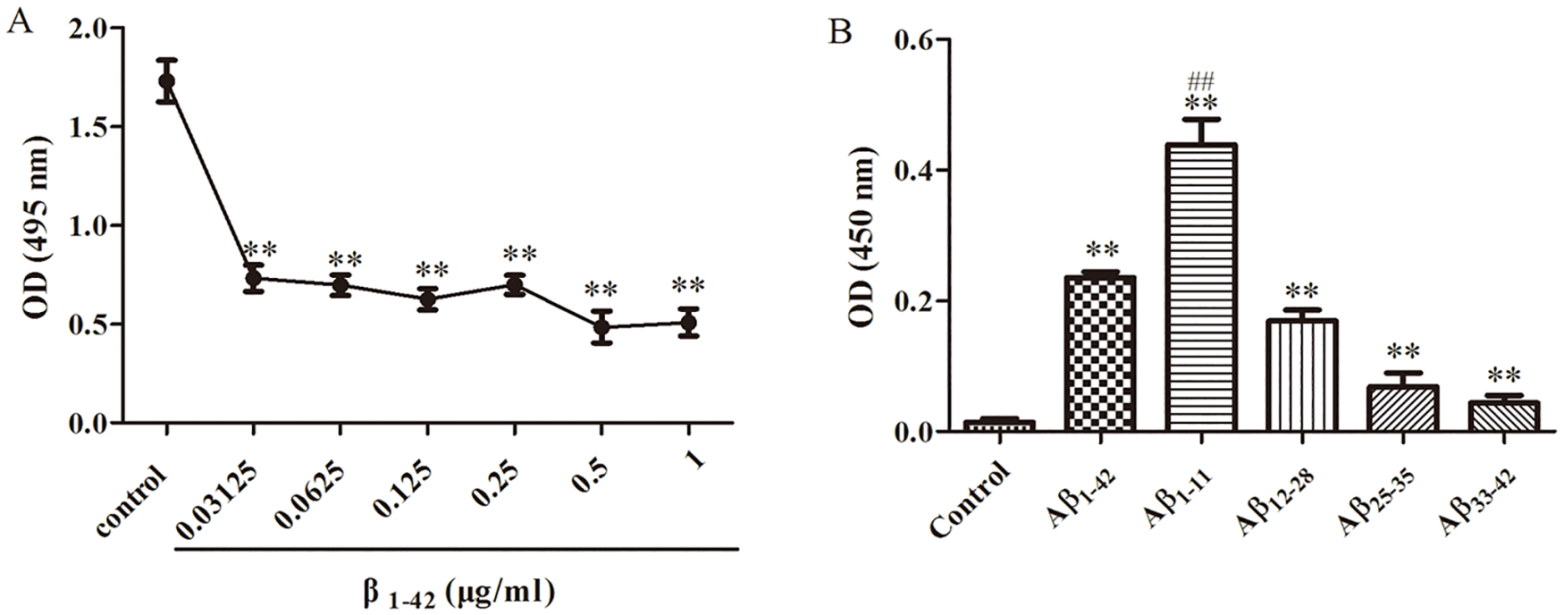
3F5 specifically binds the N-terminus of Aβ_1–42_. (A) Aβ_1–42_ (0–1 μg/mL) without FITC conjugation was pre-incubated with 3F5 overnight at 4°C. Thereafter, Aβ_1–42_ (0–1 μg/mL) without FITC conjugation and Aβ_1–42_ coated on ELISA plates competitively bound with 3F5 for 90 min at 37°C. The competitive binding of 3F5 with free or coated Aβ_1–42_ was detected by ELISA. (B) On epitope mapping, 3F5 was incubated with different fragments of Aβ_1–42_ coated on ELISA plates for 90 min at 37°C. The specificity of 3F5 for the epitopes of Aβ_1–42_ was measured by ELISA. Experiments were performed in triplicate. Data are shown as mean±SD, and analyzed by One-way ANOVA. **p < 0.01 *vs* control, ^##^p < 0.01 *vs* Aβ_1–42_ group.

As presented in Fig 1A, Aβ_1–42_ (0.03–1 μg/mL) without FITC conjugation significantly reduced the binding of 3F5 to Aβ_1–42_ conjugated with FITC. The optical density did not show significant change with the increasing dose of Aβ_1–42_ not conjugated with FITC. On epitope mapping, the OD value in the presence of Aβ_1–11_, Aβ_12–28_, Aβ_25–35_, Aβ_33–42_ and Aβ_1–42_ was markedly increased. Aβ_1–11_ showed the greatest ability to bind 3F5 than other fragments of Aβ_1–42_ (Fig 1B). These results indicate that 3F5 primarily binds the N-terminus of Aβ_1–42_.

### 3F5 protects neuronal cells from the cytotoxicity of Aβ_1–42_ fibrils

**Fig 2:**
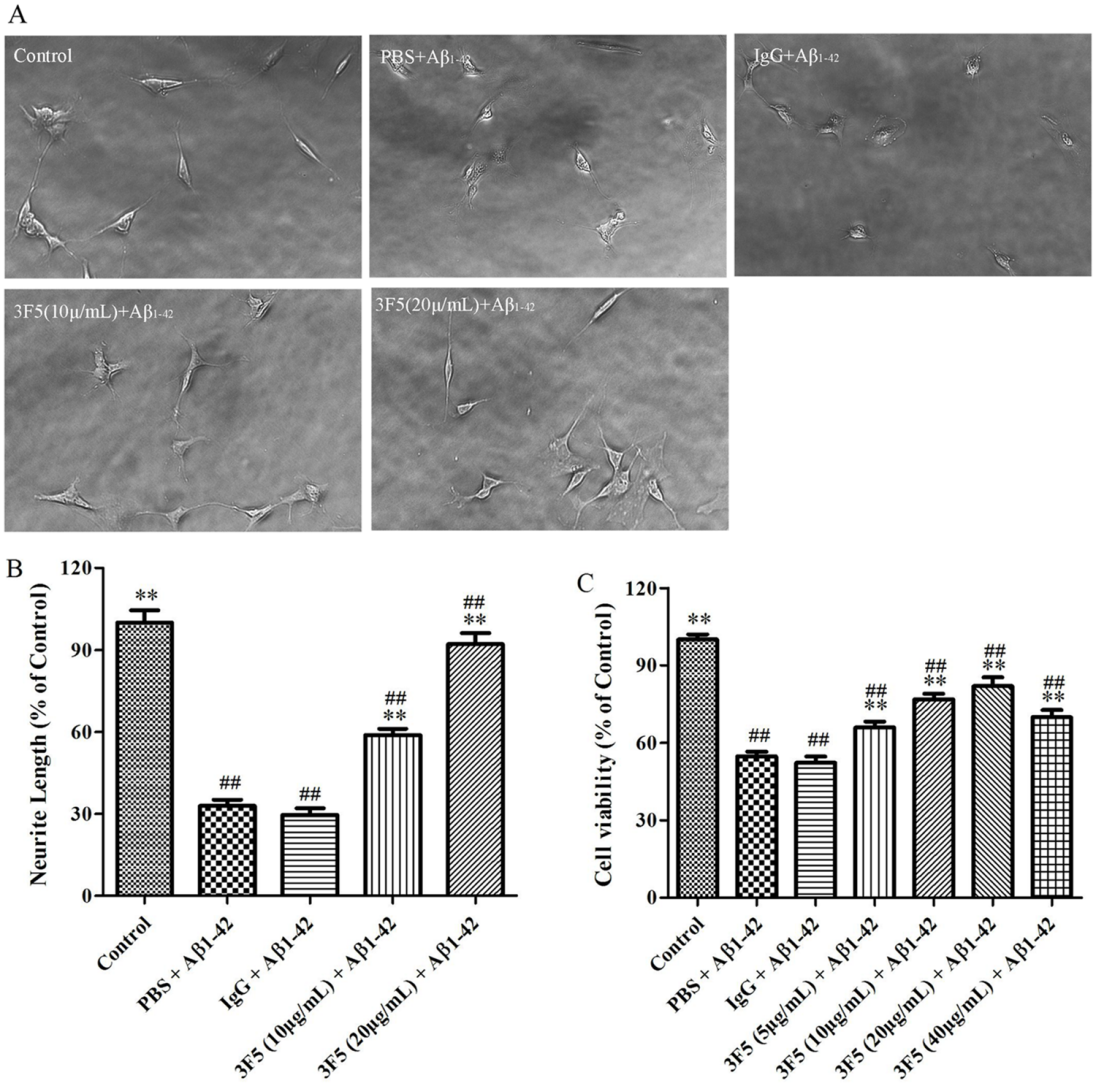
3F5 reduces the neurotoxicity of Aβ_1–42_ fibrils *in vitro*. SH-SY5Y cells were cultured with different concentrations of 3F5 and Aβ_1–42_ fibrils for the indicated times. (A) SH-SY5Y cell images were shown by microscopy (100×). (B) Quantification of neurite length for the capacity of 3F5 to stabilize neurite cells. (C) The viability of SH-SY5Y cells treated with different concentrations of 3F5 measured by MTT. Experiments were performed in triplicate. Data are shown as the mean±SD and analyzed by One-way ANOVA. **p < 0.01 *vs*. PBS+ Aβ_1–42_; ^##^*p* < 0.01 *vs*. Control.

**Fig 3:**
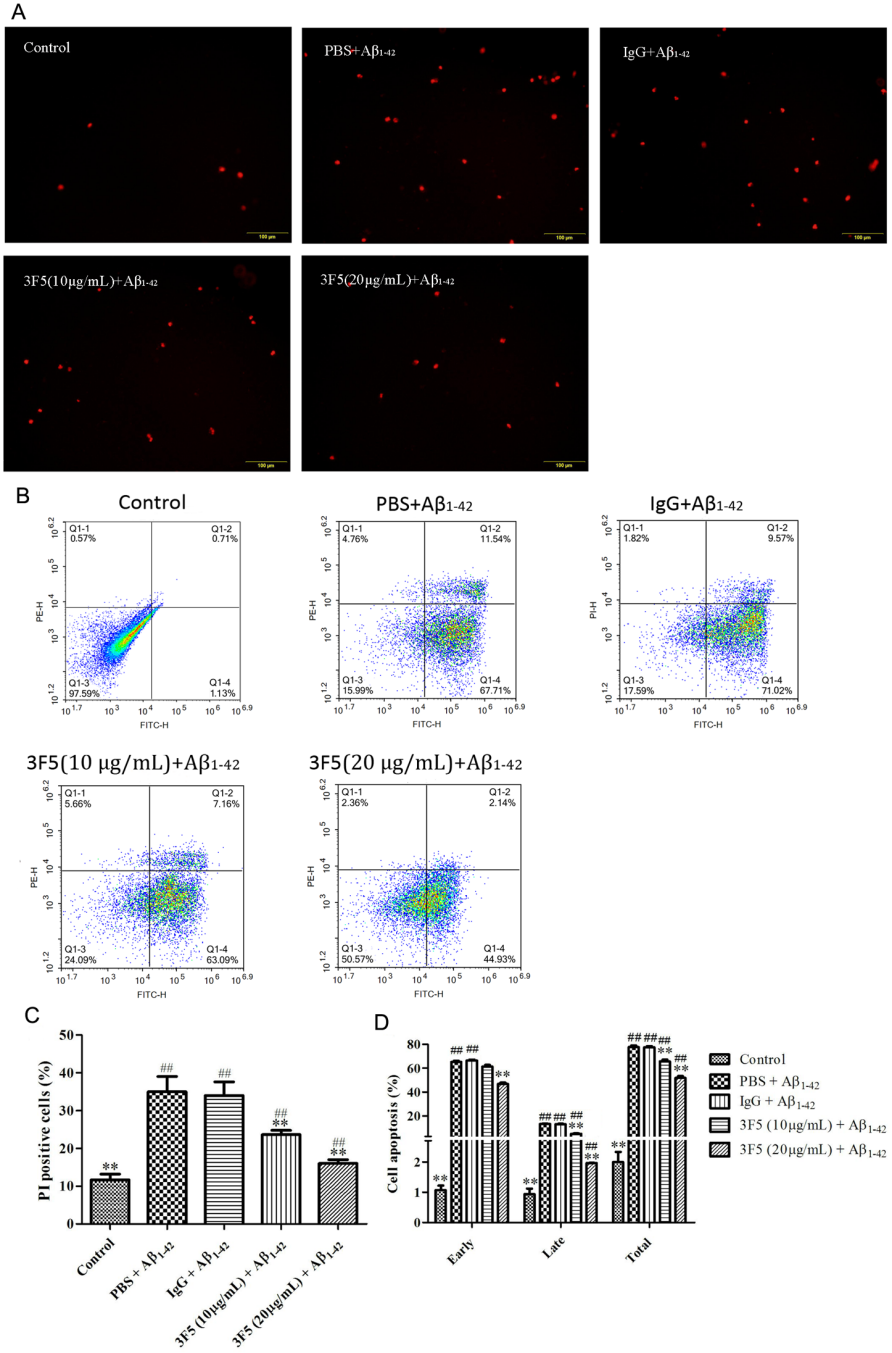
3F5 inhibits the neuronal cell apoptosis induced by Aβ_1–42_ fibrils. (A) Representative images of PI single staining to SH-SY5Y cells. SH-SY5Y cells were treated with 10 μM Aβ_1–42_ fibrils and co-incubated with PBS, IgG or 3F5 for 48 h. The Cells were then incubated with PI for 15 min in dark and analyzed with a fluorescence microscopy. (B) Representative images of SH-SY5Y cells stained with AnnexinV-FITC/PI. SH-SY5Y cells treated as above mentioned, then stained with a combination of fluorescein annexin V-FITC and PI for 10 min at room temperature. Cell apoptosis was examined with by flow cytometry. (C) The percentage of PI positive SH-SY5Y cells. (D) The percentage of cell apoptosis on SH-SY5Y cells stained with AnnexinV-FITC/PI. The experiments were repeated three times. Data are shown as the mean±SD and analyzed by One-way ANOVA. **p < 0.01 *vs*. PBS+ Aβ_1–42_; ^##^*p* < 0.01 *vs*. Control.

Aβ_1–42_ fibrils are known to be toxic for neurites. We found that the viability of SH-SY5Y cell line and the neurite length were significantly decreased in the presence of Aβ_1–42_ (Fig 2A and 2C). However, neurite length in cells treated with 3F5 (10 and 20 μg/mL) was significantly increased compared with the cells treated with Aβ_1–42_ only. 3F5 improved the viability of SH-SY5Y cells with optional concentration of 20 μg/mL (Fig 2C). Moreover, as shown in Fig 3A and 3C, 3F5 significantly reduced the late apoptosis and necrosis of SH-SY5Y cells induced with Aβ_1–42_ fibrils. 3F5 decreased the proportion of early, late and total apoptosis of SH-SY5Y cells in the presence of Aβ_1–42_ fibrils (Fig 3B and 3D). These findings confirm that 3F5 protects neurite cells from the cytotoxicity of Aβ_1–42_ fibrils.

### 3F5 rescues cognition and memory defects in Tg-mice

**Fig 4:**
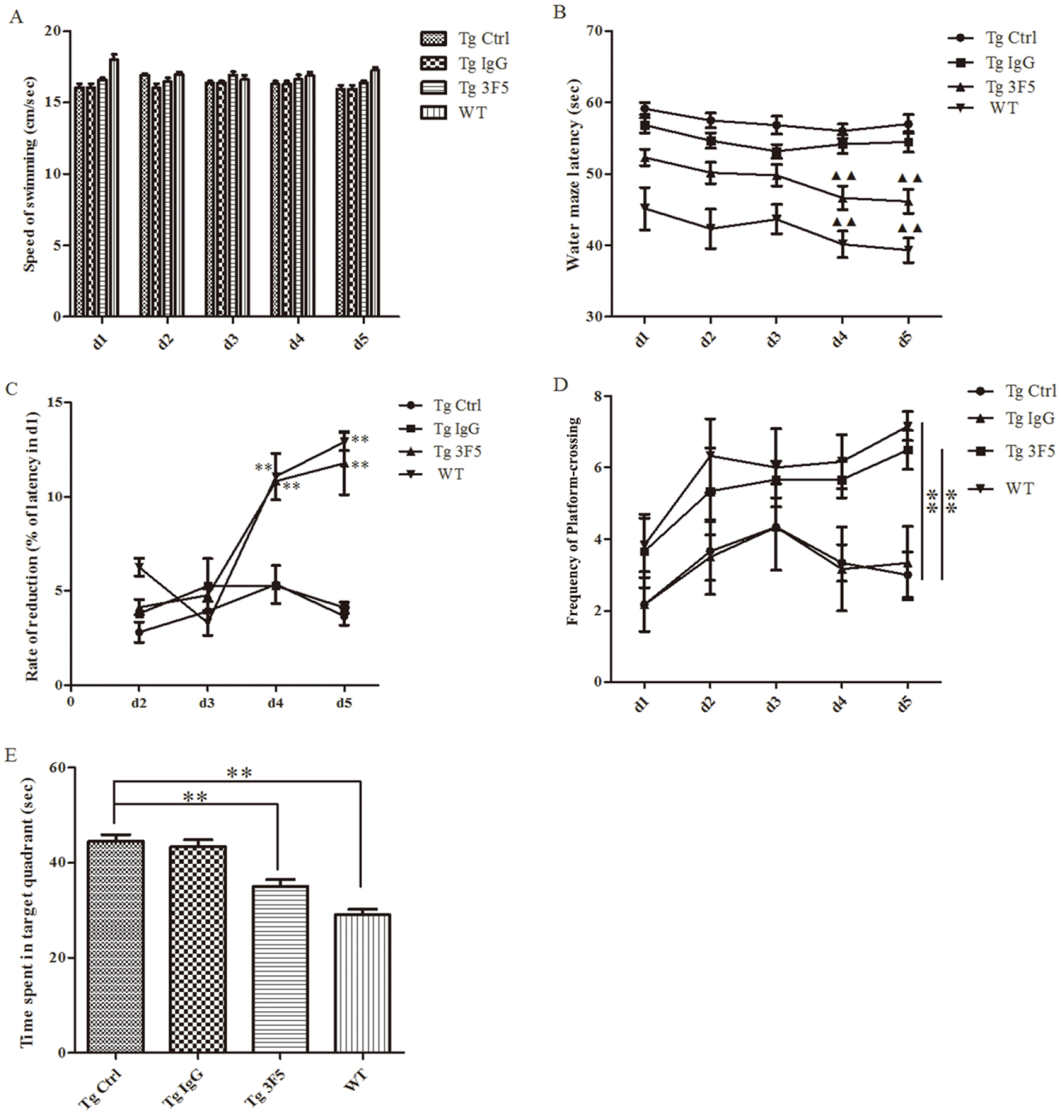
3F5 improves the cognitive and memorial performance of Tg-mice. Mice were randomly divided into 4 groups (n = 6). Mice in the 3F5-treated and IgG-treated mouse groups were injected intraperitoneally with 0.5 g/kg 3F5 or IgG twice a week for 3 months. Tg-control and WT mice were injected intraperitoneally with normal saline (NS). Morris water maze (MWM) was used to evaluate the spatial learning abilities and the memory of mice. (A) Swim speed (cm/sec) was analyzed in MWM. (B) Escape latency (sec) of mice was recorded (^▲▲^*p* < 0.01 *vs*. d1). (C) The percentage of reduction in escape latency relative to d1 was calculated. (**p < 0.01 *vs*. Tg Ctrl). (D) The frequency of platform-crossing was recorded (**p < 0.01 *vs*. Tg Ctrl). (E) Time spent in quadrants of mice was recorded (**p < 0.01 *vs*. Tg Ctrl). Tg Ctrl denotes control Tg-mice; Tg IgG denotes Tg-mice treated with IgG; Tg 3F5 denotes Tg-mice treated with 3F5. Each bar represents the mean±SD from three independent experiments, and analyzed by repeated measures ANOVA (Fig. 4A-4D) and One-way ANOVA (Fig. 4E).

Tg-mice exhibited excessive Aβ peptide deposits in the brain and defect spatial perception and memory [10]. Morris water maze test was used to assess the capacity of 3F5 to improve spatial cognition and memory of Tg-mice. Mauchly's sphericity test, tests of within-subjects effects and tests of between-subjects effects were performed. The results of Mauchly's sphericity test were *p*>0.05. As shown in Fig 4A, the swimming speed of Tg- and WT-mice was not significantly different (Tests of Within-Subjects Effects, Time, Sphericity Assumed, F = 0.618, degree of freedom (df) = 3, *p* = 0.606), indicating that mice at early stage of AD exhibited no impaired motility. However, the latency time was significantly shortened in 3F5 treated Tg-mice and WT-mice after 5 days of training (Tests of Between-Subjects Effects, Groups, F = 25.797, df = 3, *p* = 0.000). There was no apparent changes in control Tg-mice and IgG-treated Tg-mice in 5 days (Fig 4B). The reduction rate of escape latency in 3F5-treated Tg mice was similar to WT mice, and significantly prolonged than that in the Tg-control mice and the IgG-treated mice. In 3F5-treated Tg-mice, the reduction rate of escape latency reached 10.83±0.15% and 11.78±1.68% at d4 and d5 (Fig 4C, Between Groups, F = 5.328, df = 3, *p* = 0.007). WT- and 3F5-treated mice more frequently crossed the platform compared with control Tg-mice (Fig 4D, Tests of Between-Subjects Effects, Groups, F = 59.715, df = 3, *p* = 0.000). In comparison with control Tg-mice, WT- and 3F5-treated mice spent less time in the target quadrant. Under the same experimental conditions, IgG-treated Tg-mice showed no significant difference from control Tg-mice (Fig 4E). These results suggest that 3F5 reduces cognition and memory deficits in Tg-mice.

### 3F5 promotes the clearance of Aβ_1–42_ from the mouse brain

**Fig 5:**
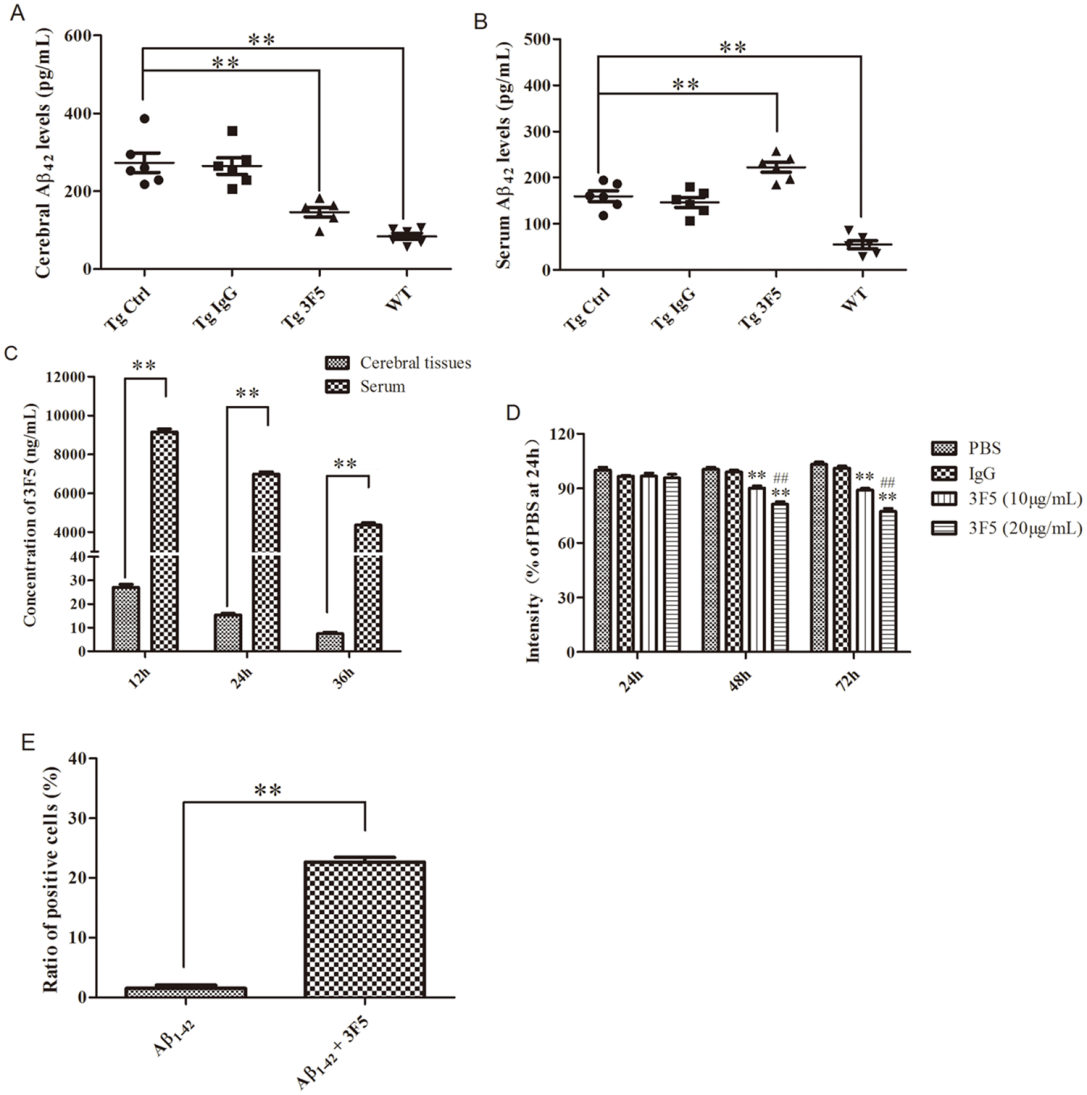
3F5 promotes the clearance of Aβ_1–42_ from the brain. Tg- and WT-mice were treated for 3-months with 3F5, IgG or NS. Aβ_1–42_ levels in the cerebral tissues (A) and in the serum (B) of Tg-mice were detected by ELISA (**p < 0.01 vs. Tg Ctrl). (C) The concentrations of 3F5 in the cerebral tissues and the serum of 3F5-treated Tg-mice were analyzed by ELISA (**p < 0.01 *vs*. cerebral tissues). (D) 3F5 (10 μg/mL and 20 μg/mL) was co-incubated with Aβ_1–42_ fibrils for the indicated times and the effect on Aβ aggregation was analyzed by ThT fluorescence (**p < 0.01 vs. PBS, ^##^*p* < 0.01 vs. 10 μg/mL of 3F5). (E) Aβ_1–42_ labelled with FITC (1 μg/mL) was co-incubated with 3F5 for 1 h followed by incubation with N9 microglial cells for 30 min at 37°C. The phagocytosis of Aβ_1–42_ by N9 microglia cells was detects by NovoCyte Flow Cytometer (**p < 0.01 *vs*. Aβ_1–42_). Tg Ctrl denotes control Tg-mice; Tg IgG denotes Tg-mice treated with IgG; Tg 3F5 denotes Tg-mice treated with 3F5. Each experiment was repeated in triplicate. Data are shown as the mean±SD and analyzed by One-way ANOVA.

To examine the capacity of 3F5 to reduce Aβ burden in the brain of Tg-mice, the concentration of Aβ_1–42_ and 3F5 in the cerebral tissues and peripheral blood were measured by ELISA. Treatment with 3F5 significantly decreased Aβ_1–42_ levels in the cerebral tissues (Fig 5A) but a marked increase Aβ_1–42_ in the serum (Fig 5B). As shown in Fig 5C, Aβ_1–42_ levels in the serum of 3F5-treated Tg-mice reached 222.32 pg/mL, which was much higher than that in the cerebral tissues of 3F5-treated Tg-mice (Fig 5A and 5B). Interestingly, the concentrations of 3F5 in the serum of 3F5-treated Tg-mice were significantly higher than that in the cerebral tissues of 3F5-treated Tg-mice (Fig 5C). Moreover, 3F5 dose-dependently reduced Aβ fibril fluorescence intensity when co-incubated with preformed Aβ fibrils for 48 and 72 h (Fig 5D). As presented in Fig 5E, after 3F5 binding with Aβ_1–42_ for 1 h, the phagocytosis of Aβ_1–42_ by murine N9 microglial cells was significantly increased. Thus, Aβ burden in the cerebral tissues was reduced by 3F5 presumably by promoting the efflux of Aβ_1–42_ from the brain, deaggregation of Aβ fibrils and phagocytosis of Aβ_1–42_ by microglia.

### 3F5 decreases the number of plaques in the hippocampus and cerebral cortex of Tg-mice

**Fig 6:**
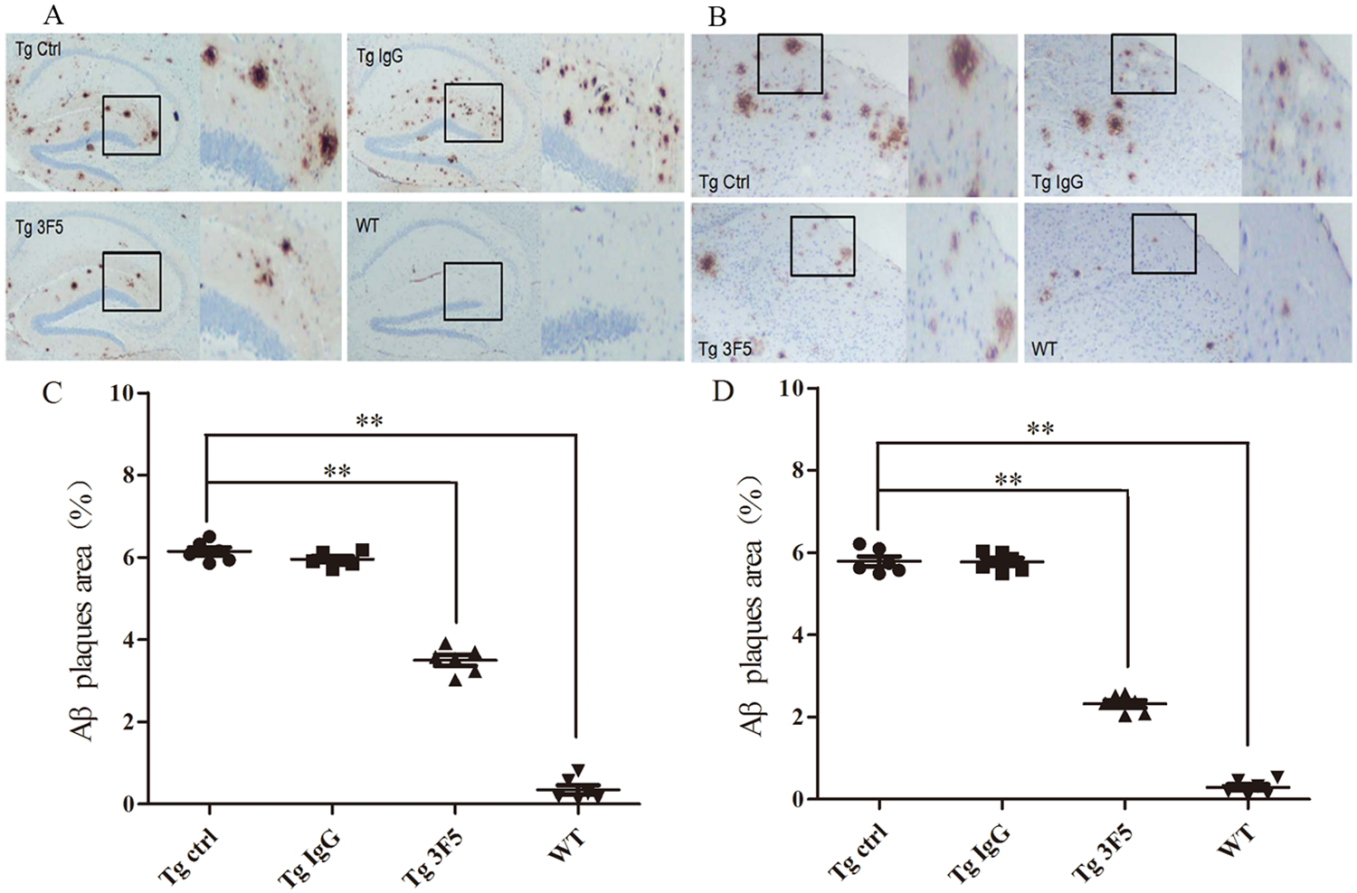
Aβ plaques in the hippocampus and cerebral cortex of mice. After 3-months treatment, the hippocampus and cerebral cortex of Tg- and WT-mice were collected for preparation of frozen sections. (A) Hippocampus sections of Tg- and WT- mice. High magnification of the deposited plaques from the boxed areas was shown. (B) Microhemorrhage associated with Aβ deposits mice in the cerebral cortex of Tg- and WT-mice (**p < 0.01 vs. the Tg-control group). (C) Aβ plaques in hippocampus area were quantitated. (D) Quantification of Aβ plaques area in the cerebral cortex (**p < 0.01 vs. the Tg-control group). Each experiment was in triplicate. Data are shown as the mean±SD and analyzed by One-way ANOVA.

Immunohistochemistry of hippocampus and cerebral cortex from Tg-mice after 3 months of antibody treatment showed (Fig 6A and 6C) Aβ plaque area in 3F5-treated mice decreased by 43% compared with the tissues from control mice which contained senior plaques mostly in the hippocampus. There were almost no senior plaques in WT mice until 9-month old (Fig 6C). IgG did not reduced Aβ plaques in Tg-mice (Fig 6A and 6C).

In agreement with Fig 6A and 6C, 3F5-treated Tg-mice exhibited 60% decrease in Aβ deposits in the cerebral cortex (Fig 6B and 6D). The number of Aβ plaques in the brain of Tg-mice and IgG-treated Tg-mice was not significantly different and there was no apparent Aβ deposition in the brains of WT mice (Fig 6D). These data demonstrate that 3F5 antibody is able to promote the clearance of senior plaques in the hippocampus and cerebral cortex of Tg-mice in association with improved cognition and memory.

### 3F5 causes minor microhemorrhage

**Fig 7:**
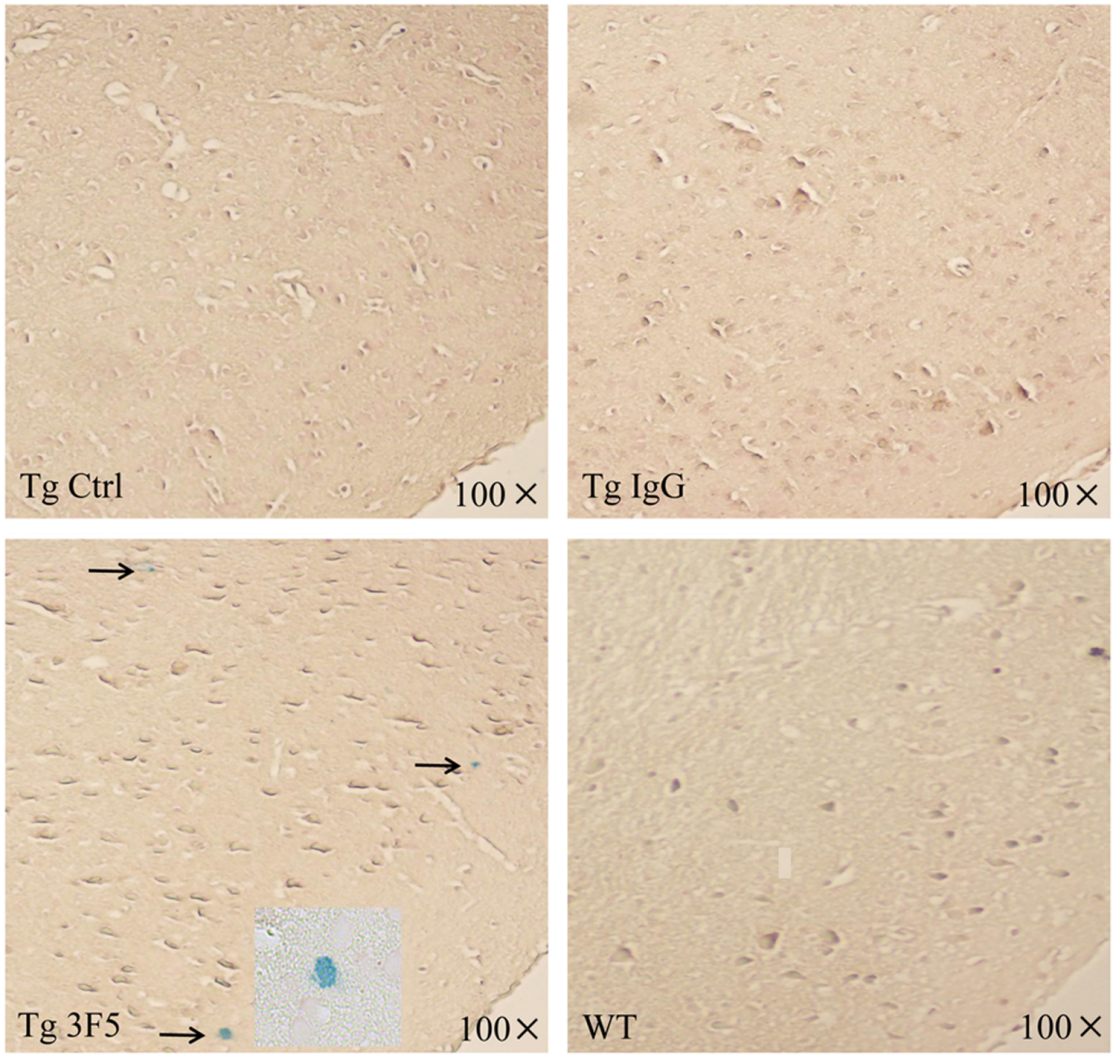
3F5 antibody increases the frequency of microhemorrhages in the brain of Tg-mice. After 3-months treatment of 3F5, sections of the cerebral tissues in Tg- and WT-mice were stained with Prussian blue and counterstained with Neutral Red. The microhemorrhage lesions were detected by the presence of hemosiderin deposits in the cerebral tissues. Images per section were acquired by Upright Metallurgical Microscopy.

Prussian blue staining was used to examine whether 3F5 antibody may increase the incidence of microhemorrhage in the brain of AD mice. As shown in Fig 7, lesions of prussian blue-positive microhemorrhage were not detected in the brain of WT-mice, control- and IgG-treated Tg-mice. However, tiny and scattered microhemorrhage lesions were detected in the cerebral tissues of 3F5-treated Tg-mice. Thus, 3F5 antibody increases lesions of microhemorrhage in the brain of 9-month-old Tg-mice, despite its beneficial effects in reverse the course of AD.

## Discussion

AD is caused mainly by progressive accumulation of Aβ_1–42_ peptide in the brain to form fibrils and amyloid plaques, which lead to synaptic loss, neuronal dysfunction and death [11]. Therefore, Aβ_1–42_ has become a major therapeutic target for AD [12]. Although anti-Aβ mAbs including bapineuzumab and solanezumab failed to show beneficial effect in clinical trials [7, 13], passive immunotherapy still offers exciting preliminary results. In this study, we successfully developed an Aβ_1–42_ N-terminus targeting antibody 3F5, and confirmed that the mAb specifically bound Aβ_1–42_ to promote depolymerization of Aβ fibrils, facilitate endocytosis of Aβ_1–42_ by microglia, and attenuate the death and apoptosis of neuronal cells, accompanied by neurite outgrowth. Moreover, therapy with 3F5 improved the behavioral abilities of Tg-mice. Further, 3F5 reduced Aβ burden and initiated Aβ efflux from the brain in Tg-mice.

Despite the development of potential therapies for AD, all double-blind, placebo-controlled Phase III studies of these therapies have failed to show clinical efficacy. Failure of all Phase III trials prompted the hypothesis that early intervention perhaps years before clinical symptoms of AD maybe crucial. [14]. Therefore, 6-month-old Tg-mice, which have not yet developed noticeable symptoms of memory loss were used in this study to investigate the therapeutic potential of 3F5. We found that 3F5 prevented memory and cognitive decline of Tg-mice, and decreased amyloid in the hippocampus and cortex. Although the accurate epitope remains "target" unclear, 3F5 may bind amino acids between 1 and 11 in the N-terminal of Aβ_1–42_ to induce depolymerization of Aβ fibrils therefore to facilitate endocytosis of Aβ_1–42_ by microglia. It is interesting to note that 3F5 preferentially bound Aβ_1–11_ *vs* Aβ_1–42_, which contains amino acids 1–11, suggesting that conformational change in Aβ_1–42_ peptide structure aggregation process may interfer its binding by 3F5 [15, 16]. The exact mechanisms of the binding of 3F5 to a conformational epitope will be investigated by analyzing the crystal structures of 3F5 complexed with Aβ.

Mounting evidence confirms the efficacy of Aβ N-terminus targeting antibodies, but side-effects, notably microhemorrhage and vasogenic edema (VE) in the brain constitute risks for AD patients [17]. Our study showed that 3F5 causes minor microhemorrhage lesions in Tg-mice accompanied with clearance of Aβ_1–42_. The finding is in agreement with a recent study showing that Bapineuzumab, a humanized IgG1, which recognizing the N terminus of Aβ (aa 4–10), clears plaques from the brains of AD patients in a phase II trial. However, it also causes cerebrovascular oedema and micro-haemorrhage in the cerebral vasculature [18, 19]. Preclinical studies suggest that Bapineuzumab may either decrease microglial response due to decreased plaque load, or enhance the cell response due to increased activation of FcγRs upon phagocytosis of IgG-Aβ complexes [20, 21]. It have been reported that modifying the backbone of the antibody may reduce FcγR affinity and inflammatory responses therefore reduce the associated side effects [3]. However, the efficacy to remove plaques and the potential to induce side effects are difficult to predict due to use of different experimental models, epitope specificity of the antibody or antibody subclass [22]. Furthermore, studies in Tg mice have not translated into successful clinical trials in humans; therefore, the response to different anti-Aβ antibodies may differ in human AD patients [22]. Therefore, larger scale trials with longer durations should be crucial for future efficacy and safety analysis of 3F5. In addition, although Aβ N-terminus-directed antibodies are more effective in dissolving preexisting Aβ plaques in the brain [23], these antibodies may also promote the formation of Aβ oligomers, which are more neurotoxic. Therefore, combination of 3F5 with other neuroprotective therapy such as anti-tau agents, gene therapies, natural compounds, and stem cells may achieve more favorable outcomes [8].

There is an equilibrium for Aβ in the brain and in the circulation [24, 25]. Our study showed that after 3F5 treatment the level of Aβ_1–42_ in the brain tissues significantly decreased in Tg-mice while there is an increase of Aβ_1–42_ in the serums. Therefore, 3F5 facilitated the efflux of Aβ_1–42_ from the brain to the circulation [26]. It is likely that free Aβ peptides in the serum may be cleared by proteases including insulin-dependent enzyme, neprilysin, α-secretase, and other nonspecific proteases if Aβ do not form aggregates resistant to degradation [27]. Interestingly, the concentration of 3F5 in the circulation is much higher than that in the brain in 3F5-treated Tg-mice (Fig 5C). Thus, 3F5 may cross BBB to bind Aβ to initiate the phagocytosis of antibody/Aβ complex by microglia. 3F5 may also conjugate with Aβ in the circulation to promote Aβ efflux from the brain and inhibit blood-to-brain influx of Aβ. This is consistent with previous study [6, 26], showing that anti-Aβ antibody may facilitate Aβ clearance through the interaction of antibody-amyloid plaque in the brain as “central clearance” and the interaction of antibody-Aβ in the circulation as “peripheral clearance” [8].

Further research is necessary to explore the efficacy, safety and functional mechanism of 3F5 in larger sizes of animals. Moreover, successful management of AD may require targeting not only Aβ but also Aβ-associated pathogenic mechanisms such as tau hyperphosphorylation, neuroinflammation, and oxidative stress [8]. Regardless, our novel anti-Aβ_1–42_ antibody 3F5 has shown potential as a basis for development of more effective therapeutic agents for AD.
